# CEMIP Promotes Osteosarcoma Progression and Metastasis Through Activating Notch Signaling Pathway

**DOI:** 10.3389/fonc.2022.919108

**Published:** 2022-07-26

**Authors:** Jun Cheng, Yan Zhang, Rongjun Wan, Jun Zhou, Xin Wu, Qizhi Fan, Jingpeng He, Wei Tan, Youwen Deng

**Affiliations:** ^1^ Department of Spine Surgery, The Third Xiangya Hospital, Central South University, Changsha, China; ^2^ Department of Respiratory Medicine, Xiangya Hospital, Central South University, Changsha, China; ^3^ Department of Dermatology, Xiangya Hospital, Central South University, Changsha, China; ^4^ Institute of Medical Sciences, Xiangya Hospital, Central South University, Changsha, China

**Keywords:** CEMIP, osteosarcoma, progression, metastasis, notch signaling pathway

## Abstract

Cell migration inducing protein (CEMIP) has been linked to carcinogenesis in several types of cancers. However, the role and mechanism of CEMIP in osteosarcoma remain unclear. This study investigated the role of CEMIP in the progression and metastasis of osteosarcoma, CEMIP was found to be overexpressed in osteosarcoma tissues when compared to adjacent non-tumor tissues, and its expression was positively associated with a poor prognosis in osteosarcoma patients. Silencing CEMIP decreased osteosarcoma cells proliferation, migration, and invasion, but enhanced apoptosis *in vitro*, and suppressed tumor growth and metastasis *in vivo*. Mechanistically, CEMIP promoted osteosarcoma cells growth and metastasis through activating Notch signaling pathway, silencing CEMIP would reduce the protein expression and activation of Notch/Jagged1/Hes1 signaling pathway *in vitro* and *in vivo*, activation of Notch signaling pathway could partially reversed cell proliferation and migration in shCEMIP osteosarcoma cells. In conclusion, our study demonstrated that CEMIP plays a substantial role in the progression of osteosarcoma *via* Notch signaling pathway, providing a promising therapeutic target in osteosarcoma.

## Introduction

Osteosarcoma is the most frequent primary malignant bone tumor occurs between the ages of 10 and 30, and peaking during the adolescent growth spurt (1, 2). The 5-year overall survival rate for osteosarcoma patients with non-metastasis is 60% to 70% (3), but sharply drops to 20% in patients with lung metastasis (4). Despite recent advances in the diagnosis and systemic chemotherapy of osteosarcoma, the prognosis for many patients remains unsatisfied due to the disease’s proclivity for recurrence and metastasis (5, 6). Numerous studies have been devoted to identifying novel targets and signaling pathways associated with osteosarcoma in order to improve the effectiveness of osteosarcoma treatment, nonetheless, the causes and molecular mechanisms underlying osteosarcoma recurrence and metastasis remain unknown (7). Thus, identifying new targets and elucidating their role and mechanisms in osteosarcoma pathogenesis are critical for the development of novel osteosarcoma therapeutic strategies.

CEMIP, alternatively referred to KIAA1199 and hyaluronan binding protein (HYBID), is a perinuclear space and cell membrane protein that is encoded by a gene on chromosome 15q25.1 (8). CEMIP has been linked to tumor progression and metastasis in different kinds of human cancers, including prostate carcinoma (9), colorectal tumors (10), hepatocellular carcinoma (11), breast carcinoma (12), gastric carcinoma (13) and oral squamous cell carcinoma (14), it acts as an oncogene, playing a vital role in the proliferation, migration and apoptosis in a variety of malignancies (15). Additionally, CEMIP is involved in the regulation of multiple signal pathways, including EMT (16), MEK/ERK (17), AMPK (18), Wnt/β-catenin (8), Ras/Raf/Erk (19) and PI3K/AKT (20), all of which are linked to the development of malignant tumors. However, the significant of CEMIP and its possible mechanism in osteosarcoma remain unknown.

Notch signaling pathway plays an important role in regulating cell proliferation, survival, apoptosis and differentiation (21), dysfunction of Notch signaling pathway impedes normal cell differentiation and causes them to transform into cancer cells, thus it has been implicated in the pathogenesis of a variety of cancers including osteosarcoma (22). CEMIP acts as an oncogene, regulating multiple signal pathways in a variety of malignant tumors, however, it remains unknown whether Notch signaling pathway has been involved in CEMIP-regulated carcinogenesis of tumors.

In this study, we found that CEMIP expression was up-regulated in tumor samples obtained from osteosarcoma patients, and its expression was associated with a poor prognosis in these individuals. Following that, the effect of CEMIP on osteosarcoma was investigated, and it was discovered that suppressing CEMIP could inhibit osteosarcoma growth and metastasis *in vitro* and *in vivo*. Finally, the possible mechanism of CEMIP promoting the osteosarcoma pathogenesis was explored, and it was demonstrated that silencing CEMIP may inhibit the carcinogenesis of osteosarcoma *via* attenuating Notch signaling pathway. Our study provides a potential target for novel osteosarcoma therapeutic strategies.

## Materials and Methods

### Tissue Samples and Cell Lines

This study was approved by the ethics committee of the Third Xiangya Hospital, Central South University (Approval No.202023), and informed consent was acquired from all patients. Six pairs of osteosarcoma tissues and adjacent non-tumor tissues were obtained from patients who underwent surgery at the Third Xiangya Hospital between October 2019 and December 2020. Additionally, paraffin-embedded tissues of osteosarcoma from 80 individuals recruited between January 2012 to December 2018 were analyzed in conjunction with their clinical characteristics including age, sex, disease stage and prognosis. Cell lines including hFOB, MG63, 143B, Saos-2, U2OS and HOS were purchased from the Chinese Academy of Science Cell Bank (Shanghai, China), and cultured in DMEM (Gibco, USA) except for Saos-2 in McCoys’5A (Gibco, USA) containing 1% penicillin/streptomycin and 10% FBS (Gibco, USA).

### shRNA Construction and Transfection

All of the shRNA were purchased from Genechem (Shanghai, China), HOS and U2OS cells were transfected according to the standard protocol, respectively. Puromycin (Beyotime, China) was used to screen the transfected cell. The shRNA sequences of CEMIP were listed in **Table 1**.

**Table 1:** The Sequences of shRNA.

ShRNA1	5’-GGTTATGACCCACCCACATAC -3′
ShRNA2	5’-GCAATCGTCCCATTGATATAC -3′
ShRNA3	5’-GGTTATGACCCACCCACATAC-3′
ShNC	5’-TTCTCCGAACGTGTCACGT-3′

### CCK-8 Assay

5000 cells were planted per well in a 96-well plate, then grown for the proper time (12, 24, 36, 48,60 and 72 hours). The cells were cultured for an additional 2 hours before the absorbance value at 460 nm was measured using CCK-8 reagent (Beyotime, China).

### EdU Assay

Cells were treated with EdU reagent (Abcam, UK) for 2 hours, then fixed in methanol and stained cell nuclei with hoechst (Abcam, UK). The EdU-positive cells were captured using fluorescence microscopy, then quantified with Image J software (NIH, USA).

### Wound Healing Assay

Cells were seeded in 6-well plates, wounded with a 200ul tip when they reached 90% saturation, and photographed at 0 and 48 hours.

### Transwell Assay

4× 10^4^ cells were seeded in the upper chambers for migration assay, and 8× 10^4^ cells were seeded in the upper chambers blocked with matrigel (BioCoat USA) for invasion assay. The top chambers were supplemented with 200μl medium containing 5% FBS (Gibco, USA), whereas the lower chambers were supplemented with 600μl medium containing 15% FBS. Cells were fixed and stained after a 24-hour incubation period, and photographed using a microscope.

### Western Blot Analysis

RIPA (NCM Biotech, China) and PMSF (NCM Biotech, China) were employed to lyse osteosarcoma cells and tissues, and BCA test were utilized to determine the concentration of protein lysates. SDS-PAGE was used to isolate proteins of various molecular masses, which were then transferred to PVDF membranes and treated with primary antibodies overnight at 4°C after 1 hour of blocking. The following day, the membrane was incubated with the appropriate secondary antibodies for 1 hour, the bands were visualized using an ECL system, and quantitated using Image J software. The antibodies used were as follows: CEMIP (1:1000, Proteintech), Bcl-2 (1:1000, Abcam), Bax (1:1000, Proteintech), MMP2 (1:1000, Proteintech), MMP9 (1:1000, Proteintech), Notch (1:1000, CST), Jagged1(1:1000, CST), Hes1(1:1000, CST), GAPDH (1:1000, CST), goat anti-mouse IgG (1:3000, CST), goat anti-rabbit IgG (1:3000, CST).

### Real-Time PCR

The PrimeScript RT reagent kit (TaKaRa, Japan) was used to reverse transcribe the RNA extracted with TRIzol (Invitrogen, USA). On an Applied Biosystems 7300 System, qPCR was done using SYBR Premix ExTaq (TaKaRa, Japan). The sequences of the mRNA primers were listed in **Table 2**.

**Table 2:** The Sequences of the mRNA Primers.

CEMIP_F	GGAGAGTTCCAAGCAGCA
CEMIP_R	CGTCAATCACCACCACCT
GAPDH_F	GGTGAAGGTCGGATGCAACG
GAPDH_R	CAAAGTTGTCATGGATGHACC

### Annexin FITC/PI Double Staining

Cells were collected and rinsed in cold PBS before being incubated with 5 μl Annexin V-FITC and 5 μl PI (Vazyme, China) at 4°C for 10 mins without light. The percentage of apoptosis cells was assessed using a BD FACS III flow cytometer (BD, USA).

### Immunohistochemistry

Each slide was deparaffinized and dehydrated prior to incubation in sodium citrate buffer, then in PBS containing 3% H_2_O_2_. It was then hatched with primary antibodies and the appropriate secondary antibodies before being incubated with the ABC solution, followed by 3,3’-diaminobenzidine (DAB) staining. Finally, the slides were xylene dehydrated and mounted with neutral glue. Photographs were taken in a light microscope. The antibodies used were as follows: CEMIP (1:100, Proteintech), Notch1 (1:100, CST), Jagged1 (1:100, CST), Hes1 (1:100, CST), goat anti-mouse IgG-FITC (1:100, CST), goat anti-rabbit IgG H&L (1:100, CST).

### H&E Staining

Each slide was deparaffinized and washed with ethanolic hydrochloric acid and ammonium hydroxide, followed by staining with eosin and dehydrating with increasing concentrations of alcohol; finally the slides were dehydrated with xylene and sealed with neutral gum.

### Animal Experiments

All animal experiments were carried out in compliance with the Experimental Animal Ethics Committee’s guidelines and were approved by the Animal Experimental Committee of the Third Xiangya hospital (Grant number: 2021sydw0221). For subcutaneous xenograft models, 1×10^6^ shCEMIP and shNC HOS cells in 200μl PBS were injected subcutaneously into BALB/c-nude mice separately. The tumor volumes were measured and calculated as previous described (23), and the mice were sacrificed after 30 days. For the metastasis model, 1×10^6^ shCEMIP and shNC HOS cells in 100μl PBS were injected into nude male mice through the tail vein. After 30 days the mice were sacrificed and the liver and lung were stained with H&E.

### Statistics Analyses

All data was described as the mean ± SD. Kaplan–Meier curves was utilized for the survival analysis. χ^2^ test was used for analyzing demographic and biological parameters. The t test was performed to evaluate the significance of a difference between different groups. The SPSS 26.0 was used for the statistical analyses, and p < 0.05 was considered as statistically significant.

## [Results]

### CEMIP Is Increased Expressed and Positively Associated With a Poor Prognosis in Osteosarcoma Patients

**Figure 1:**
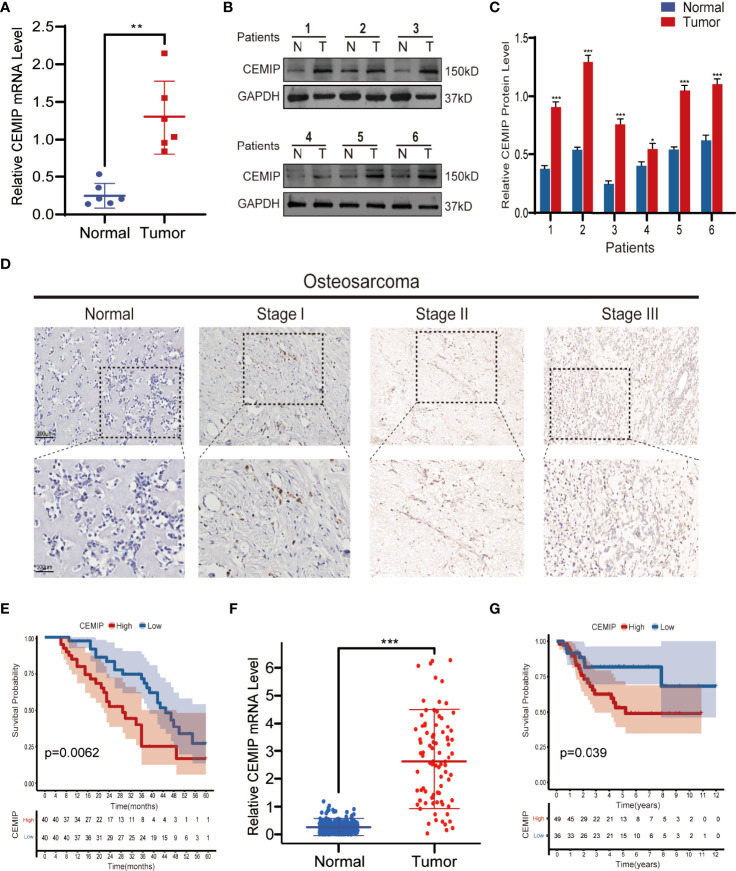
CEMIP is overexpressed in osteosarcoma tissues and related to poor prognosis. **(A)** qRT-PCR analysis of CEMIP expression in six pairs of osteosarcoma tissues. **(B, C)** Western blot and its quantitative analysis showed CEMIP was overexpressed in six paired of osteosarcoma tissues and their normal adjacent tissues. **(D)** Immunohistochemical analysis of CEMIP expression in osteosarcoma tissues at different Enneking stages. **(E)** Survival analysis of CEMIP expression in osteosarcoma patients from in-house patients. **(F)** mRNA expression of CEMIP in osteosarcoma patients from the TARGET datasets. **(G)** Survival analysis of CEMIP expression in osteosarcoma patients from the TARGET datasets. *P < 0.05, **P < 0.01, ***P < 0.001.

CEMIP has been linked to the pathogenesis of a variety of malignancies in previous studies (9–14); however, its role and mechanism in osteosarcoma remain unknown. To explore the effect of CEMIP on osteosarcoma, we detected the CEMIP expression in six pairs of osteosarcoma tissues and adjacent non-tumor tissues using qPCR and western blot, and found the CEMIP expression was significantly higher in the osteosarcoma tissues compared to the adjacent non-tumor tissues (**Figures 1A–C**). In addition, clinical data and pathology sections from eighty osteosarcoma patients were collected for IHC measurements of CEMIP expression, the results showed that increased CEMIP expression was associated with advanced osteosarcoma stage (**Figure 1D**). According to the quantity of CEMIP-positive cells, we divided these individuals into high expression group (n=40) and low expression group (n=40) averagely, the clinical characteristic of the patients were summarized and presented in **Table 3**, the data indicated that increased CEMIP expression was associated with advanced osteosarcoma stage and poorer prognosis in osteosarcoma patients (**Figure 1E**). To further confirm the role of CEMIP in osteosarcoma, the expression of CEMIP was evaluated from TARGET datasets. The result showed that the expression of CEMIP in osteosarcoma tissues was significantly higher than that in normal tissues (**Figure 1F**), and the prognosis of patients with high CEMIP expression was worse than that of patients with low CEMIP expression (**Figure 1G**), which were consistent with our findings. As a result of these data, it suggests that CEMIP expression is increased and associated with the prognosis in osteosarcoma patients.

**Table 3:** The correlation between CEMIP expression and clinical pathology parameters in OS.

Parameters	Case(n=80)	Expression		Result(p-value)
		High(n=40)	Low(n=40)	
**Age**				0.390
≥24	15	9	6	
<24	65	31	34	
**Gender**				0.329
male	56	26	30	
female	24	14	10	
**Tumor site**				0.483
femur	44	24	20	
tibia	25	10	15	
other	11	6	5	
**Tumor size**				0.098
≥3cm	34	20	14	
<3cm	46	20	26	
**Enneking stage**				0.289
I	30	13	17	
II	37	18	19	
III	13	9	4	
**Metastasis**				0.023*
No	69	31	38	
Yes	11	9	2	

*P < 0.05.

### CEMIP Promotes the Proliferation of Osteosarcoma Cells

**Figure 2:**
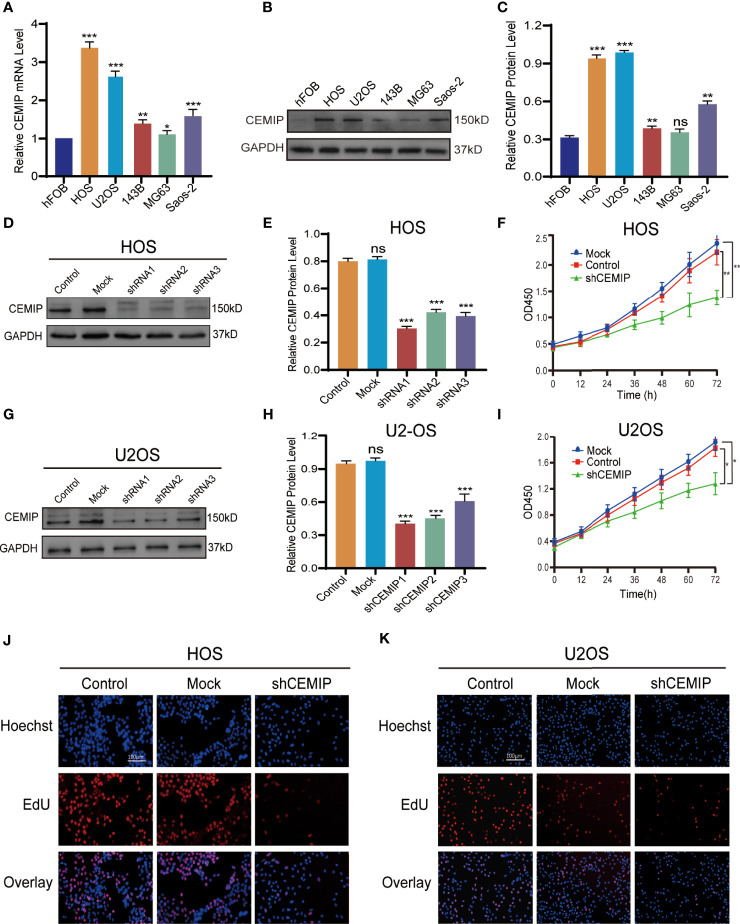
CEMIP promotes the proliferation of osteosarcoma cells. **(A)** qRT-PCR analysis of CEMIP expression in hFOB and different osteosarcoma cell lines. **(B, C)** Western blot and its quantitative expression of CEMIP in hFOB and different osteosarcoma cell lines. **(D, E)** Western blot and its quantitative expression of silencing CEMIP in HOS cells. **(G, H)** Western blot and its quantitative expression of silencing CEMIP in U2OS cells. **(F, I)** The effect of silencing CEMIP on growth of HOS and U2OS cells were measured by CCK-8 assays. **(J, K)** The effect of silencing CEMIP on growth of HOS and U2OS cells were measured by EdU assays. *P < 0.05, **P < 0.01, ***P < 0.001. ns, no significance.

To further investigate the role of CEMIP in pathogenesis of osteosarcoma *in vitro*, CEMIP expression was determined using qPCR and western blot in a panel of osteosarcoma, and the results indicated that it was most abundantly expressed in HOS and U2OS (**Figures 2A–C**), which were used in the subsequent assays. Given high expression of CEMIP in HOS and U2OS, we established CEMIP silencing osteosarcoma cells (shCEMIP group) using three kinds of shRNA and its negative control cells (Control group), and the mock group is parental osteosarcoma cells without lentivirus (Mock group). Among all three shCEMIP shRNA, shRNA-1 had the greatest silencing effect in both HOS and U2OS when the CEMIP expression was detected using western blot (**Figures 2D–E, G–H**), thus cells transfected with shRNA-1 were selected as the shCEMIP group for further studies. Following that, the ability of osteosarcoma cells to proliferate was determined using CCK-8, and the result showed cell proliferation was approximately same in the mock group and control group, but considerably slower in the shCEMIP group (**Figures 2F, I**). Meanwhile, the EdU assay was used to examine the cell proliferation, and it yielded comparable results as well (**Figures 2J, K**). These findings indicated that CEMIP could promote osteosarcoma cells proliferation *in vitro*.

### CEMIP Promotes the Invasion and Migration of Osteosarcoma Cells

**Figure 3:**
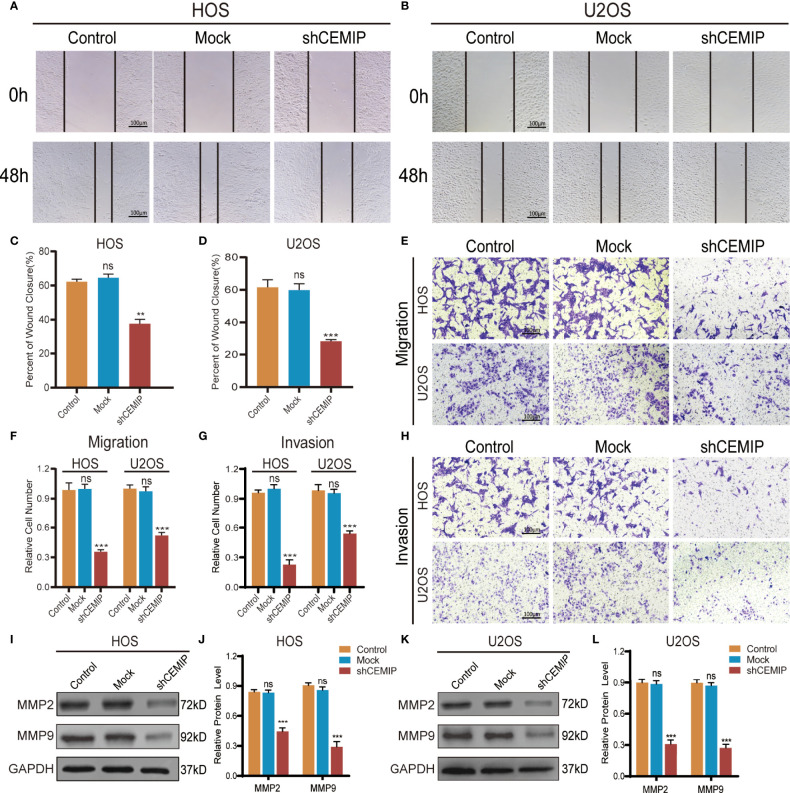
CEMIP promotes the invasion and metastasis of osteosarcoma cells. **(A, C)** Cell migration capacity in HOS were detected by wound healing assays and their quantitative analysis. **(B, D)** Cell migration capacity in U2OS were detected by wound healing assays and their quantitative analysis. **(E, F)** Cell migration ability in HOS and U2OS cells were analyzed by transwell migration assays and their quantitative analysis. **(G, H)** Cell invasion ability in HOS and U2OS cells were analyzed by transwell invasion assays and their quantitative analysis. **(I, J)** Western blot and the relative protein expression of MMP2 and MMP9 in HOS cells. **(K, L)** Western blot and the relative protein expression of MMP2 and MMP9 in U2OS cells. **P < 0.01, ***P < 0.001. ns, no significance.

To elucidate the role of CEMIP in osteosarcoma pathogenesis, wound healing assays, transwell assays were conducted as well. In the wound healing assay, silencing CEMIP resulted in a reduction of cell migration when compared to the mock and control groups (**Figures 3A–D**). Transwell assays including migration assay and invasion assay revealed that the shCEMIP group had considerably fewer cells that migrated or penetrated through the membrane than the mock and control groups (**Figures 3E–H**). Matrix metalloproteinase (MMP) is a type of enzyme that promotes tumor cells metastasis, and MMP-2 and MMP-9 are commonly used as tumor metastasis indicators. MMP-2 and MMP-9 expression were significantly inhibited by shCEMIP group (**Figures 3I–L**) when compared to the mock and control group. As a result of these findings, we concluded that CEMIP could promote the invasion and migration of osteosarcoma cells *in vitro*.

### CEMIP Suppresses Cell Apoptosis in Osteosarcoma

**Figure 4:**
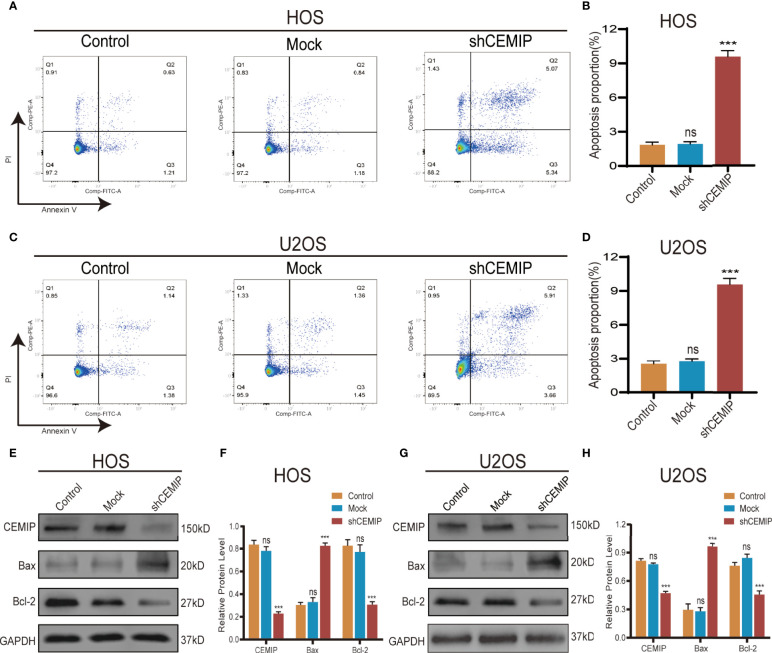
CEMIP knockdown induces cell apoptosis. **(A, B)** The role of silencing CEMIP on apoptosis of HOS cells were detected by flow cytometry assays and its quantitative analysis. **(C, D)** The role of silencing CEMIP on the apoptosis of U2OS cells were detected by flow cytometry assays and their quantitative analysis. **(E, F)** Western blot showed the relative protein expression of apoptosis markers (pro-apoptotic marker, Bax; anti-apoptotic marker, Bcl-2) in HOS cells. **(G, H)** Western blot showed the relative protein expression of apoptosis markers (pro-apoptotic marker, Bax; anti-apoptotic marker, Bcl-2) in U2OS cells. ***P < 0.001. ns, no significance.

Flow cytometry was used to investigate the effect of CEMIP on cell apoptosis in HOS and U2OS, and the results revealed that the shCEMIP group had a larger proportion of apoptosis cells than the mock and control groups (**Figures 4A–D**). Additionally, Bax expression, indicator of apoptosis, was up-regulated, while anti-apoptotic protein Bcl-2 was suppressed in the shCEMIP group when compared to mock and control groups (**Figures 4E–H**). These data indicated that CEMIP could suppress apoptosis of osteosarcoma cells *in vitro*.

### Silencing CEMIP Inhibits the Growth and Metastasis of Osteosarcoma *In Vivo*

**Figure 5:**
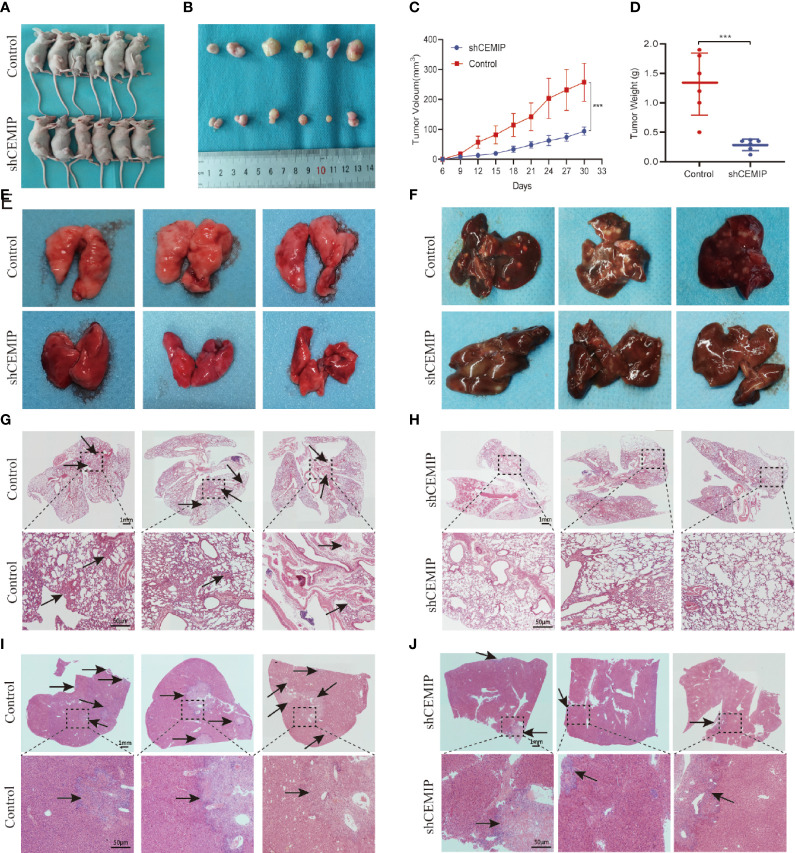
CEMIP promotes the growth and metastasis of osteosarcoma in vivo. **(A)** Representative images of xenograft models. **(B)** Representative images of xenograft tumors from respective groups. **(C)** Tumor growth curves from respective groups. **(D)** Average tumors weight from respective groups. **(E)** Representative images of the lungs of nude mice from metastsis models. **(F)** Representative images of the livers of nude mice from metastsis models. **(G, H)** Histological features of lung metastases by H&E staining. **(I, J)** Histological features of liver metastases by H&E staining. ***P < 0.001.

In order to further determine the role of CEMIP in pathogenesis of osteosarcoma cells *in vivo*, HOS cells from shCEMIP group and control group were injected subcutaneously into nude mice respectively. Tumor volume was measured every three days and the mice were sacrificed after 30 days, The results indicated that, in comparison to the control group, mice in shCEMIP group had smaller tumor size and lower weight (**Figures 5A–D**). Additionally, in the metastasis models, although neither group had visible metastatic lesions on the lung surface (**Figure 5E**), both nude mice groups had visible metastatic tumors on the liver surface (**Figure 5F**). Moreover, H&E staining of the lungs revealed glimpsed lung metastasis lesions in the control group (**Figure 5G**), but considerably less in the shCEMIP group (**Figure 5H**). In the livers, H&E staining showed the control group had more and larger metastatic lesions (**Figure 5I**) than the shCEMIP group (**Figure 5J**). Thus, our findings indicated that silencing CEMIP would be able to suppress osteosarcoma growth and metastasis *in vivo*.

### CEMIP Promotes the Progression of Osteosarcoma Through Notch Signaling Pathway

**Figure 6:**
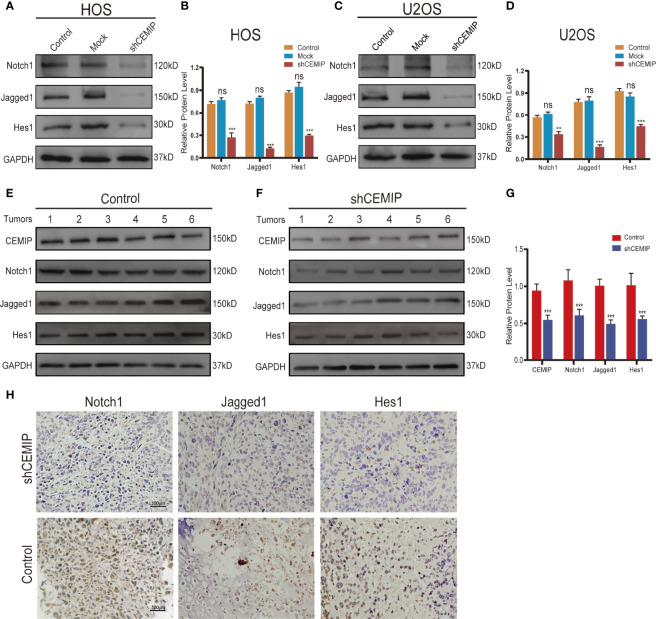
CEMIP promotes the growth and metastasis through Notch signaling pathway. **(A, B)** Western blot and their quantitative analysis of silencing CEMIP on expression of Notch1, Jagged1 and Hes1 in HOS cells. **(C, D)** Western blot and their quantitative analysis of silencing CEMIP on expression of Notch1, Jagged1 and Hes1 in U2OS cells. **(E–G)** Western blot and their quantitative analysis of CEMIP, Notch1, Jagged1 and Hes1 in tumor tissues from two groups of xenograft tumors. **(H)** Immunohistochemical analysis of Notch1, Jagged1 and Hes1 in osteosarcoma tissue from two groups of xenograft tumors. **P < 0.01, ***P < 0.001. ns, no significance.

As showing in the results, we found that silencing CEMIP significantly decreased the protein expression of Notch receptor Notch1, Notch ligand Jagged1, and Notch target gene Hes1 in osteosarcoma cells (**Figures 6A–D**). In addition, we used western blot and immunohistochemistry to assess Notch1, Jagged1 and Hes1 expression in tumor tissues from nude mice, and the results demonstrated a reduced expression of Notch/Jagged1/Hes1 signaling pathway in tumor tissues from the shCEMIP group in comparison to the control group (**Figures 6D–G**).

**Figure 7:**
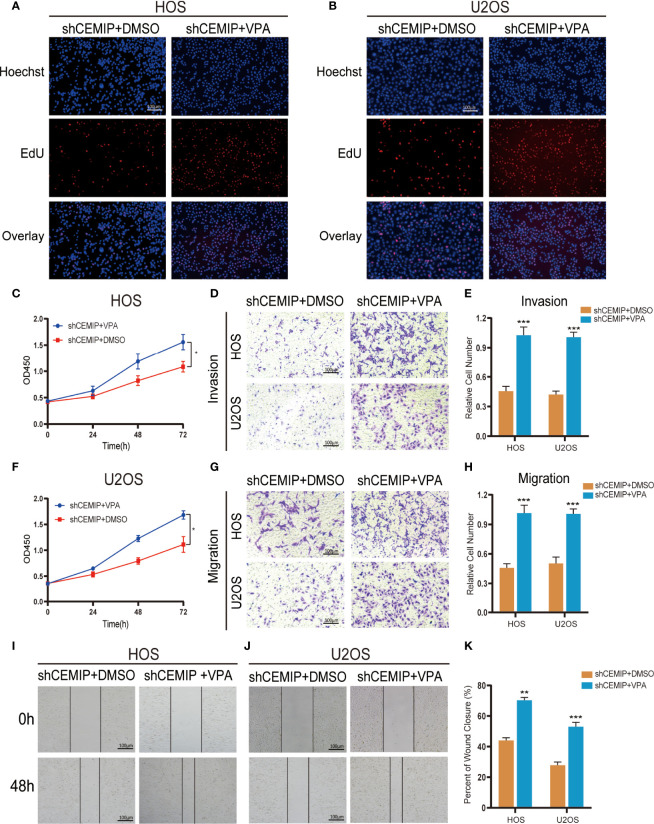
Activation of Notch signaling pathway reverses growth, invasion and migration of osteosarcoma cells caused by siliencing CEMIP. **(A, B)** The effect of VPA on growth of shCEMIP osteosarcoma cells were measured by EdU assays. **(C, F)** The effect of VPA on growth of shCEMIP osteosarcoma cells were measured by CCK-8 assays. **(D, E)** The effect of VPA on invasion of shCEMIP osteosarcoma cells were analyzed by transwell invasion assays and their quantitative analysis. **(G, H)** The effect of VPA on migration of shCEMIP osteosarcoma cells were analyzed by transwell migration assays and their quantitative analysis. **(I–K)** The effect of VPA on migration capacity of shCEMIP osteosarcoma cells were detected by wound healing assays and its quantitative analysis. *P < 0.05, **P < 0.01, ***P < 0.001.

To further confirm the regulatory role of Notch signaling pathway in CEMIP-regulated osteosarcoma cells growth and metastasis, we stimulated shCEMIP osteosarcoma cells using Valproic acid, a Notch signaling activator (VPA), which was dissolved in DMSO. The EdU assay revealed that VPA can promoted cells proliferation in shCEMIP HOS and shCEMIP U2OS cells (**Figures 7A, B**), the same results were presented in the CCK8 assays (**Figure 7C, F**). Transwell assays including invasion assay and migration assay revealed that VPA could promote cells migrate or penetrate through the membrane in shCEMIP HOS (**Figures 7D, E**) and shCEMIP U2OS (**Figures 7G, H**). Meanwhile, the wound healing assay revealed that VPA promoted shCEMIP HOS and shCEMIP U2OS migration as well (**Figures 7I–K**). Taken together, our findings revealed that CEMIP could promote osteosarcoma progression and metastasis through activating Notch signaling pathway *in vitro* and *in vivo*.

## Discussion

In the current study, we found that CEMIP was overexpressed in osteosarcoma tissues, which was positively correlated with advanced tumor stage and poor prognosis of the disease. Mechanically, silencing CEMIP dramatically reduced osteosarcoma cells proliferation, invasion and migration *in vitro*, inhibited osteosarcoma cells growth and metastasis *in vivo*, and increased the proportion of apoptotic cells. Additionally, we demonstrated that Notch signaling pathway was involved in the CEMIP-regulated pathogenesis of osteosarcoma. Therefore, we not only investigated the relationship between CEMIP expression and clinical significance of osteosarcoma, but also revealed that CEMIP could promote the proliferation and metastasis and suppress cell death of osteosarcoma cells, thus leading to the malignant progression of osteosarcoma.

CEMIP was first discovered with an unknown function in 1999 (24), four years later, Satoko Abe identified it as an inner ear-specific protein since its genetic abnormalities were linked to non-syndromic hearing loss (25), CEMIP exists in normal human tissues with low expression level, such as brain, pancreas and testis. It was first discovered to be overexpression in tumor cells by Michishita in 2006 (9), since then, CEMIP as a marker of high metastatic potential and poor prognosis has been shown in different malignancies (10–14). Despite the fact that there are many studies on CEMIP and tumors, the studies on CEMIP and musculoskeletal tumors are still quite few. Koike found the forced expression of CEMIP effectively suppressed the tumorigenicity of low‐grade chondrosarcoma with abundant hyaluronan (26). Recently, high expressions of CEMIP and hyaluronan were reported to be as poor prognostic factors for osteosarcoma, but in which no verification or exact mechanism were clarified (27). Given the complex link between CEMIP and musculoskeletal tumors, therefore, additional research on the role of CEMIP in musculoskeletal tumors is extremely desirable. Thus, in the current study, we not only investigated the relationship between CEMIP expression and clinical significance of osteosarcoma, but also revealed that CEMIP could promote the proliferation and metastasis and suppress cell death of osteosarcoma cells, thus leading to the malignant progression of osteosarcoma.

The feature of easy recurrence and metastasis pose a considerable challenge for the clinical treatment of osteosarcoma, resulting in no effective treatment in the advanced stage of the disease (7). Our results provided substantial evidence that CEMIP is an oncogenic and metastatic risk for osteosarcoma, suggesting that targeting CEMIP may provide a novel strategy for osteosarcoma treatment. As an oncogene, CEMIP does more than merely enhance cell proliferation and metastasis, it also plays a vital role in the drug resistance of tumor cells. Studies showed that CEMIP was essential for maintaining cell metastasis and EMT in sorafenib-resistant hepatocellular carcinoma cells (16), and also demonstrated that CEMIP trigged acquired resistance to selumetinib in colorectal cancer cells (17). Furthermore, CEMIP promotes tumor cell adaptation to a harsh microenvironment, Evensen et al. discovered that up-regulation of CEMIP is a critical regulator of colon cancer cells dissemination in a hypoxic microenvironment, hypoxia-inducible-factor-2*α* (HIF-2*α*) binds directly to the hypoxia response element within the CEMIP promoter region, resulting in increased CEMIP expression and enhanced cell migration (28). Additionally, it was reported that CEMIP could induce tumor angiogenesis *via* interacting with the EGFR pathway of cervical tumors in a NF-κB dependent manner (29). Besides, Terashima M revealed that upregulating CEMIP could lead to high tumor incidence by boosting the gluconeogenesis for energy (30), and Liu et al. observed that upregulating CEMIP resulted in increased transcription of SLC7A11 in prostate cancer cells by increasing cystine absorption, thereby promoting ferroptosis resistance in cells detached from the extracellular matrix (31).

According to our findings, CEMIP can promote osteosarcoma cells proliferation and metastasis *via* Notch signaling pathway. Notch signaling pathway is an evolutionarily conserved signaling system which has been implicated in the pathogenesis of a variety of diseases (23); dysfunction of Notch signaling pathway impairs cell differentiation and induces malignant transformation, leading to tumor angiogenesis, stemness of cancer cells, and EMT (32). Notch pathway is indispensable for skeletal development, however, when it is disrupted, abnormal osteoblast and osteoclast production occurs, leading to skeletal disease (33). According to current studies, notch target genes and notch receptors are overexpressed in osteosarcoma, and govern osteosarcoma cells proliferation and differentiation (34). It has been confirmed that Notch ligand Jagged1 was overexpressed in osteosarcoma (35), and activation of Notch signaling pathway will promote the occurrence, progression and metastasis of osteosarcoma (36–39), as well as to chemotherapy resistance (40, 41). In this study, we revealed that Notch signaling pathway is involved in CEMIP-regulated osteosarcoma, and CEMIP promotes osteosarcoma cells growth and metastasis *in vitro* and *in vivo* through activating Notch signaling pathway.

Although the function of CEMIP in osteosarcoma was intensively investigated, there are still some shortcomings in this study. First, we did not further study the downstream target genes that were directly regulated by CEMIP in osteosarcoma. Second, we found that activation of the Notch signaling pathway could partially reverse the proliferation and metastasis of shCEMIP osteosarcoma cells but not completely, which indicated that there are other signaling pathways involved in CEMIP-regulated osteosarcoma cells, this result reflected that CEMIP could modulate tumor progression in multiple ways. Last but not the least was that, in our metastatic models, the main metastatic site is the liver instead of the lung, which was a very unique phenomenon that was different from previous studies, we did not delve into the mechanism of this phenomenon in the current study, but exploring the possible mechanism would be warranted in our follow-up studies.

In summary, our study indicated that CEMIP was over-expressed in osteosarcoma, and correlated with the prognosis of osteosarcoma patients. CEMIP promoted osteosarcoma cells progression and metastasis through activating Notch signaling pathway, thus suggesting that CEMIP could be a potential target for gene therapy of osteosarcoma.

## Data Availability Statement

The datasets presented in this study can be found in online repositories. The names of the repository/repositories and accession number(s) can be found in the article/supplementary material.

## Ethics Statement

The studies involving human participants were reviewed and approved by The Ethics Committee of The Third Xiangya Hospital. The patients/participants provided their written informed consent to participate in this study. The animal study was reviewed and approved by the Animal Experimental Committee of the Third Xiangya hospital. Written informed consent was obtained from the individual(s) for the publication of any potentially identifiable images or data included in this article.

## Author Contributions

JC, WT and YD designed the research. JC and JZ performed the research. XW and QF analyzed the data from Target datesets. JC and YZ wrote the paper. JC and JH organized the original source data. All authors reviewed the paper and approved the final version.

## Funding

Science and Technology Program of Hunan Province, Award Numbers:2021RC4057. Postgraduate Scientific Research Innovation Project of Hunan Province.

## Conflict of Interest

The authors declare that the research was conducted in the absence of any commercial or financial relationships that could be construed as a potential conflict of interest.

## Publisher’s Note

All claims expressed in this article are solely those of the authors and do not necessarily represent those of their affiliated organizations, or those of the publisher, the editors and the reviewers. Any product that may be evaluated in this article, or claim that may be made by its manufacturer, is not guaranteed or endorsed by the publisher.
